# Record of *Phlebotomus (Transphlebotomus) mascittii* Grassi, 1908 and *Phlebotomus (Larroussius) chadlii* Rioux, Juminer & Gibily, 1966 female in Algeria

**DOI:** 10.1051/parasite/2011184337

**Published:** 2011-11-15

**Authors:** Z. Berdjane-Brouk, R.N. Charrel, I. Bitam, B. Hamrioui, A. Izri

**Affiliations:** 1 Parasitologie-Mycologie, CHU Avicenne, Université Paris 13 Bobigny France; 2 Unité des Virus Émergents (UMR190), Université de la Méditerranée – Institut de Recherche pour le Développement Marseille France; 3 Laboratoire d’Entomologie Médicale, Institut Pasteur d’Alger Algérie; 4 Laboratoire de Parasitologie, Hôpital Mustapha Alger Algérie

**Keywords:** *Phlebotomus mascitti*, *Phlebotomus chadlii*, Algeria, *Phlebotomus mascitti*, *Phlebotomus chadlii*, Algérie

## Abstract

We report for the first time the presence of *Phlebotomus mascittii* and the female of *Phlebotomus chadlii* in Algeria. These two species were collected during an entomological study conducted in endemic visceral leishmaniasis focus from the north part of the country, Kabylia.

## Abstract (fr)

Les auteurs signalent pour la première fois la présence de *Phlebotomus mascittii* et de la femelle de *Phlebotomus chadlii* en Algérie. Ces deux espèces ont été capturées lors d’une enquête entomologique réalisée dans un foyer de leishmaniose viscérale du nord du pays, la Kabylie.

Twenty-two phlebotomine sand fly species (Diptera: Psychodidae) have been reported in Algeria, 12 belonging to the *Phlebotomus* genus and 10 to the *Sergentomyia* genus (Belazzoug, 1991). Those included in the *Phlebotomus* genus are of medical importance since they comprise recognized or suspected vectors of leishmaniasis and/or Phlebovirus. We report here for the first time (i) the presence of *Phlebotomus mascittii* in Algeria, and (ii) the presence of the female *Phlebotomus chadlii* in the same area.

The entomological investigation was conducted in Larbaa Nath Iraten (4° 12’ 05’’ E, 36° 38’ 10’’ N at 916 m altitude), in a humid bioclimatic zone, in Kabylian area (Izri *et al.*, 2008). Sand flies collection was performed during summer 2009 using CDC miniature light traps.

A total of 883 sand flies (703 males and 180 females) were captured and morphologically identified during 16 night-CDC traps (55 sand flies/night-CDC traps). Ten distinct species were identified: one species belonging to the *Sergentomyia* genus (*S. minuta*) and nine species to the *Phlebotomus* genus including one female of *P. mascittii* and two females of *P. chadlii* (Table 1).

**Table 1.** Sand fly species diversity in LNI, Kabylian area during summer 2009).

Specie	Male	Female	Total
*P. (Larroussius) perniciosus*	564	115	679
*P. (Larroussius) longicuspis*	84	39	123
*P. (Larroussius) langeroni*	24	0	24
*P. (Larroussius) perfiliewi*	3	4	7
*P. (Larroussius) ariasi*	1	2	3
*P. (Larroussius) chadlii*	1	2	3
*P. (Paraphlebotmus) sergenti*	3	7	10
*P. (Phlebotomus) papatasi*	1		1
*P. (Transphlebotomus) mascittii*		1	1
*S. (Sergentomyia) minuta*	22	10	32

Total	703	180	883

*P. mascittii* was described in Italy (Roma), then in other countries in the north shore of the Mediterranean basin, from Spain to Turkey (Seccombe *et al.*, 1993). In countries of northern Europe, it was reported in Germany and Switzerland (Naucke *et al*., 2000). However, *P. mascittii* has always been found in low density. In France, *P. mascittii* species was observed in several departments including in the north, such as Alsace (Callot, 1950). In southern regions, it was usually associated with the main recognized vectors of visceral leishmaniasis, *P. ariasi* and *P. perniciosus* (Rioux *et al.*, 1984; Pesson *et al.*, 1985). It was described as an anthropophylic and aggressive species (Pesson *et al.*, 1985). *P. mascittii* was suspected to be a vector of Mediterranean leishmaniasis, because it was frequently collected from human and dog leishmaniasis in endemic foci (Pesson *et al.*, 1985). However, its vector role has not been confirmed so far. Hence, we noticed for the first time the presence of *P. mascittii* female (Fig. 1) in the southern part of Mediterranean. This female was collected from animal shelter localized in house basement.

**Fig. 1.**
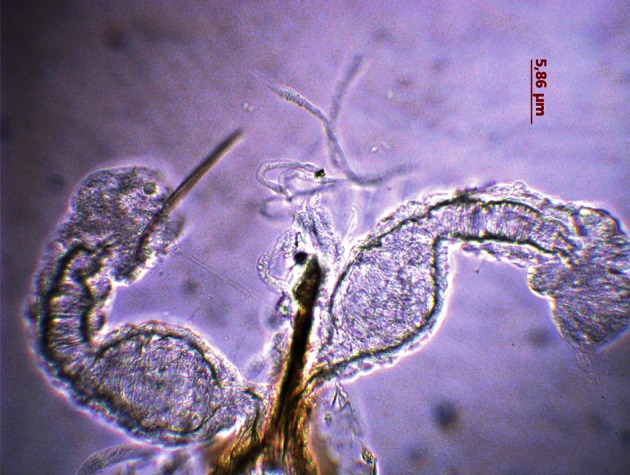
Spermathecae of *Phlebotomus (Transphlebotomus) mascitti* (photonic microscope × 200).

*P. chadlii* was described from Northwest Tunisia (El Kef) among male sand fly specimens. However, the female remained unrecognized until 2006 when it was described in specimens trapped in El Kef, Tunisia (Chamkhi *et al.*, 2006). In Algeria, *P. chadlii* is widely spread in humid, sub humid and arid bioclimatic zones (Dedet *et al.*, 1984). For unknown reasons, in Algeria, only male specimens have been reported so far (Rioux *et al.*, 1970; Dedet *et al.*, 1984). In our survey, three specimens of *P. chadlii* were identified, two females (Fig. 2) and one male, all of them cached in animal shelters. The bioclimatic distribution of *P. chadlii* coincides with that of *P. ariasi* (Dedet *et al.*, 1985), the proven vectors of *L. infantum* and of Sand fly Fever *Phleboviruses* (SFV) in the Mediterranean basin (Izri *et al.*, 2008). Using the mitochondrial *cyt b* gene, Franco *et al.* (2010) reported that *P. chadlii* might be a sister group of the European and the Moroccan *P. ariasi* species. However, to date there is no confirmation neither for their vector role, nor for their trophic preferences. The two females collected in this study were not engorged, thus precluding blood meal analysis. We strongly support the idea of further studies (i) to elucidate the relationship between *P. chadlii* and *P. ariasi*, (ii) to identify their trophic preferences, and (iii) to study the relationship host/leishmaniasis parasite.

**Fig. 2.**
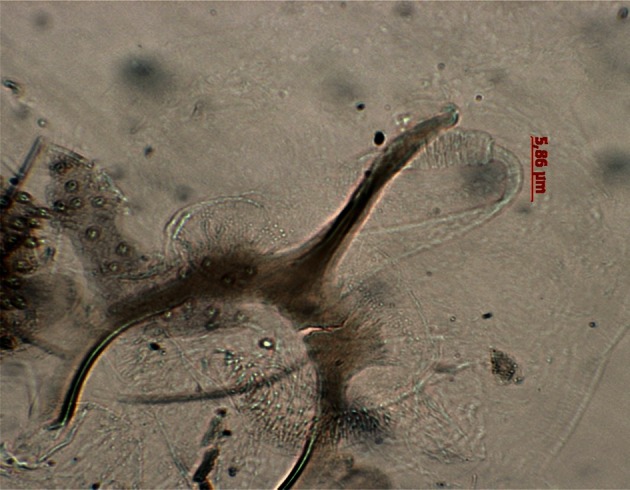
Spermathecae of *Phlebotomus (Larroussius) chadlii* (photonic microscope × 200).
